# Antimicrobial activity and mode of action of 1,8-cineol against carbapenemase-producing *Klebsiella pneumoniae*

**DOI:** 10.1038/s41598-021-00249-y

**Published:** 2021-10-21

**Authors:** Chew-Li Moo, Mohd Azuraidi Osman, Shun-Kai Yang, Wai-Sum Yap, Saila Ismail, Swee-Hua-Erin Lim, Chou-Min Chong, Kok-Song Lai

**Affiliations:** 1 Department of Cell and Molecular Biology, Faculty of Biotechnology and Biomolecular Sciences, Universiti Putra Malaysia, 43400 Serdang, Selangor Malaysia; 2 Health Sciences Division, Abu Dhabi Women’s College, Higher Colleges of Technology, 41012 Abu Dhabi, United Arab Emirates; 3 Faculty of Applied Sciences, UCSI University, No. 1, Jalan Menara Gading UCSI Height, Cheras, 56000 Kuala Lumpur, Malaysia; 4 Department of Microbiology, Faculty of Biotechnology and Biomolecular Sciences, Universiti Putra Malaysia, 43400 Serdang, Selangor Malaysia; 5 Aquatic Animal Health and Therapeutics Laboratory, Institute of Bioscience, Universiti Putra Malaysia, 43400 Serdang, Selangor Malaysia

**Keywords:** Drug discovery, Microbiology

## Abstract

Antimicrobial resistance remains one of the most challenging issues that threatens the health of people around the world. Plant-derived natural compounds have received considerable attention for their potential role to mitigate antibiotic resistance. This study was carried out to assess the antimicrobial activity and mode of action of a monoterpene, 1,8-cineol (CN) against carbapenemase-producing *Klebsiella pneumoniae* (KPC-KP). Results showed that resazurin microplate assay and time-kill analysis revealed bactericidal effects of CN at 28.83 mg/mL. Zeta potential showed that CN increased the surface charge of bacteria and an increase of outer membrane permeability was also detected. CN was able to cause leakage of proteins and nucleic acids in KPC-KP cells upon exposure to CN and ethidium bromide influx/efflux experiment showed the uptake of ethidium bromide into the cell; this was attributed to membrane damage. CN was also found to induce oxidative stress in CN-treated KPC-KP cells through generation of reactive oxygen species which initiated lipid peroxidation and thus damaging the bacterial cell membrane. Scanning and transmission electron microscopies further confirmed the disruption of bacterial cell membrane and loss of intracellular materials. In this study, we demonstrated that CN induced oxidative stress and membrane damage resulting in KPC-KP cell death.

## Introduction

*Klebsiella pneumoniae,* one of the Gram-negative opportunistic pathogens is often the cause of hospital-acquired infections; these include bloodstream infections, respiratory and urinary tract infections, especially in immunocompromised patients with underlying diseases such as chronic pulmonary obstruction, diabetes mellitus or cancer^1,2^. It has been reported that there is high mortality rate in immunocompromised patients infected by multi-drug resistant *K. pneumoniae* isolates, ranging between 18 and 49%^3^. Over the years, *K. pneumoniae* producing extended-spectrum β-lactamases (ESBLs) has become widespread throughout the world, becoming a critical threat to human health. Plasmid-mediated ESBL that is able to hydrolyze extended-spectrum cephalosporins, a class of β-lactam antibiotics derived from the mold *Cephalosporium acremonium,* was first discovered in 1983^4,5^. The first generation cephalosporins were active against Gram-positive bacteria while second generation agents have increased activity against Gram-negative bacilli. Third generation cephalosporins were significantly more active against Gram-negative bacilli compared to second generation, while the fourth generation cephalosporins were effective against both Gram-positive and Gram-negative bacteria^6^.

Due to increased use, cephalosporin resistance was developed, and carbapenems were utilized in their place. Of the many different β-lactams, carbapenems possess the widest spectrum of activity and are the most potent against both Gram-positive and Gram-negative bacteria^7^. Carbapenems are the last line of β-lactam antibiotics proven to be effective in severe infections caused by ESBL producing bacteria^8^. Continuous reliance on carbapenems further led to increasing carbapenem resistance as *K. pneumoniae* isolates started to produce the enzyme carbapenemase, to hydrolyze the carbapenems. *K. pneumoniae* carbapenemase (KPC) which is encoded by the gene *bla*KPC, is located within Tn4401, a Tn3-type transposon; it has the ability to insert itself into diverse plasmids of Gram-negative bacteria. Due to horizontal gene transfer, the enzyme has also been found in other Gram-negative bacteria such as *Escherichia coli, Salmonella enterica* and *Pseudomonas aeruginosa*^2^. It has been estimated that the third generation cephalosporins and carbapenems would be inefficacious to treat serious infections caused by *K. pneumoniae* around the world by 2030^9^. In order to address the issue of antibiotic resistance, there is a need for constant efforts to discover and develop newer antimicrobial agents, especially those of plant origin.

Plants produce complex secondary metabolites with a plethora of compounds which may have potential application as new antimicrobials^10–12^. Terpenes are the largest class of secondary metabolites fundamentally consisting of five carbon isoprene units linked to each other in many different ways; they are made up of simple hydrocarbons, while terpenoids are the modified version of terpenes with different functional groups and oxidized methyl group either removed or moved at different positions^13^. The number of carbon units categorizes terpenoids into hemiterpenes, monoterpenes, diterpenes, sesquiterpenes, sesterpenes and triterpenes^14^. Plant-derived compounds have gained considerable amount of attention due to their important physiological and ecological role in plants, as well as their uses in pharmaceutical and industrial applications including flavors, fragrances and medicines^15^.

Most of the monoterpenes exhibit strong antibacterial activities^16^. Zacchino et al.^17^ has reported that of the potentiators of antibacterial drugs streptomycin, gentamicin, nafcillin, tobramycin and amikacin, 75% are monoterpenes and diterpenes. Besides that, α-terpineol, α-pinene and linalool were proven to have antibacterial activities in *E. coli* O157:H7^18^. Another study by Silva et al.^19^ demonstrated that linalool possessed antibacterial activity against *Staphylococcus aureus* and *P.aeruginosa* with the minimum inhibitory concentration (MIC) of 1024 µg/mL. The sensitivity of bacteria towards terpenoids are determined by the composition, charge of the outer structures and membrane permeability of the bacteria. Monoterpenes will most likely alter the bacterial membrane permeability and increase membrane fluidity which causes the change in the topology of membrane proteins, giving rise to the interruption in the respiratory process^20^. Besides that, it is suggested that the lipophilicity and/or hydrophobicity and presence of hydroxyl group in the terpenes affect the antibacterial mechanism^18^.

The bicyclic monoterpene 1,8-cineol (CN), commonly known as eucalyptol, can be found in various essential oils such as the eucalyptus oil, rosemary oil and *C. longepaniculatum* leaf essential oil^21,22^. CN is used in the treatment of inflammatory diseases of the respiratory system such as sinusitis, chronic obstructive pulmonary disease and bronchial asthma due to its anti-inflammatory property^23^. Several studies reported that CN also exhibits antibacterial activity against *K. pneumoniae*, *E. coli*, *Salmonella enteritidis*, *S. aureus*^21,24^. According to Sokovicx́ et al.^25^, CN was effective against human pathogenic bacteria strains, namely *Bacillus subtilis*, *E. coli* O157:H7, *Enterobacter cloacae*, *Micrococcus flavus*, *P. aeruginosa*, *Proteus mirabilis*, *S. aureus*, *S. enteritidis*, *Staphylococcus epidermidis*, and *Salmonella typhimurium* with the MIC value ranging from 4.0 to 7.0 µg/mL. Despite the known anti-inflammatory property and antibacterial activity of CN against a few types of mentioned bacteria, scientific data regarding the antibacterial mechanism of CN against bacteria is limited, especially with reference to antibiotic resistant bacteria to assess the antimicrobial activity of CN against KPC-KP cells. Thus, this study aimed to elucidate the mechanism of action of CN against KPC-KP cells via membrane-related assays, and oxidative stress assessment which further confirmed with scanning electron microscopy and transmission electron microscopy.

## Materials and methods

### Compound

1,8-cineol used in this study was purchased from Merck KGaA (Darmstadt, Germany).

### Bacterial strain and growth conditions

The bacterial strain *K. pneumoniae* BAA-1705 (KPC-KP) was purchased from American Type Culture Collection (ATCC, Manassas, VA, USA). KPC-KP is Modified Hodge Test positive, *K.pneumoniae* carbapenemase producer and *bla*KPC positive. KPC-KP is resistant to meropenem, imipenem and ertapenem. The bacterium was grown and cultured on Mueller–Hinton agar (MHA; Sigma-Aldrich). A single colony was inoculated into Mueller–Hinton broth (MHB; Sigma-Aldrich) and incubated at 37 °C, with constant shaking at 250 rpm for 16 h.

### Resazurin microplate assay

Broth microdilution was performed to determine the minimum inhibitory concentration (MIC) of CN in KPC-KP. This assay was performed as previously described with slight modification^26^. MHB incorporated with Tween 80 at the final concentration of 10% was used to enhance CN solubility in MHB. Resazurin, a dye, at the final concentration of 0.03% was used to aid the visualization. Twofold dilution was performed in a 96-well plate. All wells consisted of 50 µL of CN, 40 µL of bacterial suspension at approximately 1 × 10^5^ CFU/mL and 10 µL of 0.03% resazurin. The 96-well plate was incubated at 37 °C at 200 rpm for overnight. The MIC of CN was determined qualitatively and quantitatively based on the observation in colour change of resazurin. The assay was performed in triplicate.

### Time-kill analysis

An inoculum of 1 × 10^5^ CFU/mL KPC-KP was used in the time-kill analysis through viable colony forming unit count. The test concentrations of CN used were the MIC (28.83 mg/mL) and half of the MIC (14.42 mg/mL). Treatment was divided into two groups, an untreated group (inoculum with MHB supplemented with 10% (w/v) of Tween 80 as final concentration) and treated-group treated with CN. Each treatment had a final volume of 20 mL incorporated supplemented with final concentration of 10% (w/v) Tween 80 to enhance the solubility of CN. Samples were incubated at 37 °C with continuous shaking at 200 rpm. Immediately after inoculation, viable counting was performed every 2 h for the first 8 h, and subsequently every 4 h for 24 h. For CFU determination, samples were serially diluted with 0.85% (w/v) sodium chloride, then 20 µL of each diluted sample was plated onto MHA. The colonies were counted after the incubation of plates at 37 °C for 16 h. This analysis was performed in triplicate.

Only half MIC of CN was used throughout the study to ensure that sufficient amount of cells could be collected to perform the assays and experiments. CN-treated KPC-KP cells at MIC (28.83 mg/mL) often resulted in no or too little growth of cells which was insufficient to proceed with the assays and experiments.

### Zeta potential measurement

Surface charge of CN-treated and untreated KPC-KP was determined using Zetasizer Nano ZS instrument (Malvern Insruments, Malvern, UK). Treatment time was based on time-kill analysis while concentration of CN was half of the MIC dose. Treated cells were washed with 0.85% (w/v) saline solution for at least three times before zeta potential measurement. This experiment was carried out in triplicate, performed as previously described with slight modification^27^.

### Outer membrane permeability assay

Outer membrane permeability assay was performed as detailed previously with slight modification^25^. Overnight culture of KPC-KP cells at OD_600nm_ 0.3 (Spectrophotometer 6300, Jenway) were subjected to 14.42 mg/mL CN with treatment time determined from the resazurin microplate assay and time-kill analysis. The samples were washed with 0.85% (w/v) saline five times upon completion of the treatment in order to remove the treatment. The remainder was divided into two equal portions of 10 mL. Following this, sodium dodecyl sulfate (SDS, Merck,USA) solution at a final concentration of 0.1% (w/v) was added to one of the portions whereas the other portion was added with 0.85% (w/v) saline. SDS acts as a permeabilizing probe that causes cell death when sudden influx occurred. This can be measured in terms of absorbance (OD_6oonm_) at intervals of 0, 5, 10, 30 and 60 min via a spectrophotometer (Spectrophotometer 6300, Jenway). The assay was completed in triplicate.

### Measurement of UV-absorbing materials

Measurement was performed as described with slight modification^28^. The untreated and 14.42 mg/mL CN-treated KPC-KP cells culture were incubated at 37 °C with continuous shaking at 200 rpm for 12 h. Cells were harvested by centrifugation (8000*g*, 10 min, 4 °C). Absorbance of the supernatants was taken at 260 nm and 280 nm to measure the content of nucleic acid and protein respectively in an extracellular environment. This experiment was carried out in triplicate.

### Ethidium bromide influx/efflux assay

Ethidium bromide (EtBr) accumulation and efflux were assessed by a Tecan microplate reader (Tecan Trading AG, Switzerland). This assay was performed as detailed previously with slight modification^29^. During the accumulation assay, KPC-KP cells were grown in MHB with continuous shaking at 37 °C until the optical density value of 0.6 was obtained. Cells were then collected and centrifuged. The cell pellet was washed and resuspended with 0.85% (w/v) sodium chloride. Bacterial suspension was then adjusted to a final value of 0.3 at OD_600nm_ with 0.85% (w/v) sodium chloride. KPC-KP cells were divided into three groups; one group consisted of untreated KPC-KP and two groups with 14.42 mg/mL CN-treated KPC-KP cells. EtBr was added to all the groups to yield a final concentration of 1 mg/L. With the excitation wavelength of 530 nm, detection wavelength of 585 nm, readings were taken every 5 min for 60 min. Next, in the efflux assay, cells were centrifuged, and the supernatant removed and resuspended with 0.85% (w/v) sodium chloride solution. Glucose with the final concentration of 0.6% (w/v) was added with or without CN to the cell resuspended in 0.85% (w/v) sodium chloride solution. The fluorescence was measured at every 5 min for 60 min. This experiment was performed in triplicate.

### Measurement of reactive oxygen species (ROS)

Measurement of ROS was performed as detailed by Kumar et al.^30^ with slight modification. KPC-KP cells were cultured in MHB and treated with 14.42 mg/mL CN. Cells were centrifuged at 10,000 rpm for 5 min and washed twice with phosphate buffered saline (PBS). This was followed by resuspending the cell pellets in fresh MHB containing 20 μM of 2′,7′-dichlorofluorescein diacetate (DCF-DA) dye in PBS to each well of a 96-well plate and incubated for 30 min at 37 °C. The stock DCF-DA stock (10 mM) was prepared by dissolving 5 mg of DCF-DA powder in 1 mL of dimethyl sulfoxide. The working stock (20 μM) was prepared by dissolving 20 μL of DCF-DA stock to 10 mL PBS buffer. The preparation of stock and working suspension was performed in the dark and only prepared immediately before use. After the incubation, medium consisting DCF-DA was decanted and 200 μL of fresh PBS was added to each well. The fluorescence intensity was measured using a Tecan microplate reader (Tecan Trading AG, Switzerland) at an excitation and emission wavelengths of 485 and 528 nm respectively. Fold change between treated and untreated cells was determined. The assay was completed in triplicate.

### Measurement of lipid peroxidation

Measurement of lipid peroxidation in cell media and cell lysate was performed as detailed by Kumar et al.^30^ with slight modification. KPC-KP cells were treated with 14.42 mg/mL CN for 12 and pelleted by centrifugation at 10,000 rpm for 5 min, washed with PBS. The resulting supernatant was collected and referred to as treatment media. The pellet was sonicated on ice, at 20 amplitude for 10 cycles. Each cycle contained 10 s of sonication followed by a 20 s cooling period (Qsonica Sonicator, Fischer Scientific, USA). The ultra-sonicated samples were then centrifuged at 4 °C and 10,000 rpm for an hour. The resulted supernatant was collected and named as cell lysate. The treatment media and cell lysate were subjected to malondialdehyde (MDA) measurement by mixing 500 μL of either treatment media or cell lysate to 400 μL of 15% (v/v) trichloroacetic acid and 800 μL of 0.67% (w/v) thiobarbituric acid in 0.01% (v/v) butylated hydroxytoluene. The samples were vortexed and incubated for 20 min at 95 °C in a water bath. The solution was allowed to cool, and 3 mL of butanol was added to the samples and gently mixed. A volume of 200 μL of the upper phase of the solution (butanol phase) was collected to take readings. The absorbance readings were measured at 532 nm. MDA level in the test samples was calculated by comparing with MDA standard curve and expressed in equivalent MDA after normalization with total protein. Total protein was measured using the Bradford assay. This assay was performed in triplicate.

### Scanning electron microscopy

This analysis was performed as detailed previously with slight modification^31^. KPC-KP cells were treated with 14.42 mg/mL of CN and treatment time were determined from time-kill analysis. The harvested cells were washed with 0.85% (w/v) saline for five times. Washed samples were fixed with 4% glutaraldehyde and 1% osmium tetroxide for 5 h and 2 h respectively at 4 °C. In all the washing steps, 0.1 M sodium cacodylate buffer was used. Next, the samples were sequentially exposed to increase acetone concentrations (35–95%) to further dehydrate the samples for 10 min followed by 100% acetone for 15 min for three times. This was followed by subjecting the samples to critical point drying for 30 min (BalTec CPD, 030, Bal-Tec, Balzers, Liechtenstein). Double sided taped was then used to secure the samples onto the specimen stub. Finally, a cool sputter coater (BalTec SCD 005) was used to sputter-coat the samples with gold and observed by a JEOL JSM-6400 instrument (JEOL, Tokyo, Japan) at 15 kV.

### Transmission electron microscopy

This analysis was performed as detailed previously with slight modification^32^. KPC-KP cells were treated with 14.42 mg/mL of CN and treatment time were determined from time-kill analysis. The harvested cells were washed with 0.85% (w/v) saline for five times. Washed samples were fixed in 4% glutaraldehyde for 2 days at 4 °C. Next, samples were washed with 0.1 M sodium cacodylate buffer three times for 30 min. Samples were post-fixed in 1% osmium tetroxide for 2 h at 4 °C and then washed again for three times with 0.1 M sodium cacodylate buffer for 30 min. This was followed by dehydration where the samples were washed with increasing acetone concentration starting at 35%, 50%, 75% and 95% for 45 min in each washing. Lastly, 100% acetone was used in the last washing step which took an hour and repeated for three times. Acetone and resin mixture were used to infiltrate the samples at the ratio of 1:1 for 12 h, 1:3 for 12 h and 0:1 for 16 h. Then, the samples were embedded into beam capsules which had been filled with resin mixture followed by polymerization process in the oven at 60 °C for 48 h.

After polymerization, the sample blocks were cut into 1 μm thick sections and stained with toluidine blue and dried on a hot plate. Excess stain was cleared by washing under running tap water. The samples were viewed under a light microscope and areas of interest were subjected to ultrathin sectioning. Resin block was trimmed according to selected area of interest by shaving the resin blocks into a trapezoid having the dimensions without exceeding 0.5 mm across he based, 0.4 mm across the top and 0.3 mm along the sides^32^. Ultrathin sectioning was completed by cutting the trapezoid block using i-Ultramicrotome EM UC6 (Leica, Germany) and samples were collected from the surface of the water bath and set on copper meshes. The samples were then stained with uranyl acetate for 15 min and washed with distilled water followed by staining with lead for 10 min and washed with distilled water^32^. Finally, the samples were viewed under a transmission electron microscope (TEM) Leo Libra-120 (ZEISS, Germany).

### Statistical analysis

All results represent the average of three independent experiments. The data were presented as mean ± standard deviation and analyzed by the Student's t-test. A *P* < 0.05 was considered as significant.

## Results

### Resazurin microplate assay and time-kill analysis

**Figure 1:**
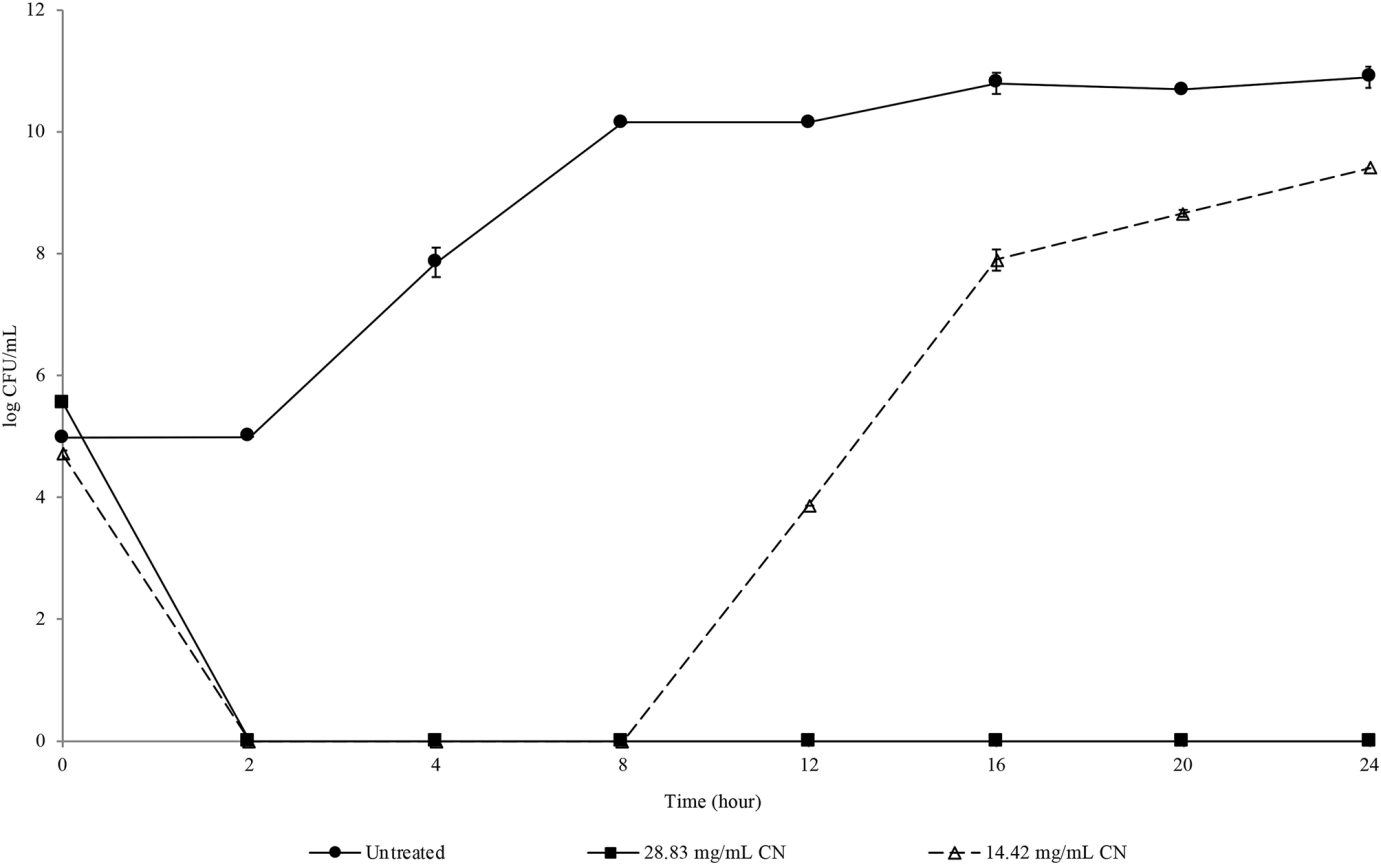
Killing kinetics of KPC-KP treated with CN at 28.83 mg/mL and 14.42 mg/mL for 24 h.

The antibacterial activity of CN was first assessed against KPC-KP via broth microdilution with the use of resazurin and time-kill analysis. CN demonstrated bactericidal activity against KPC-KP at MIC of 28.83 mg/mL. Time-kill analysis was performed to determine the killing kinetics of CN and the optimum treatment time for subsequent experiments. As shown in Fig. 1, a complete killing profile of CN-treated KPC-KP at MIC was obtained within 2 h. CN-treated KPC-KP cells at half MIC were killed and regrowth was observed at the 12th h.

### Outer membrane permeability assay

**Figure 2:**
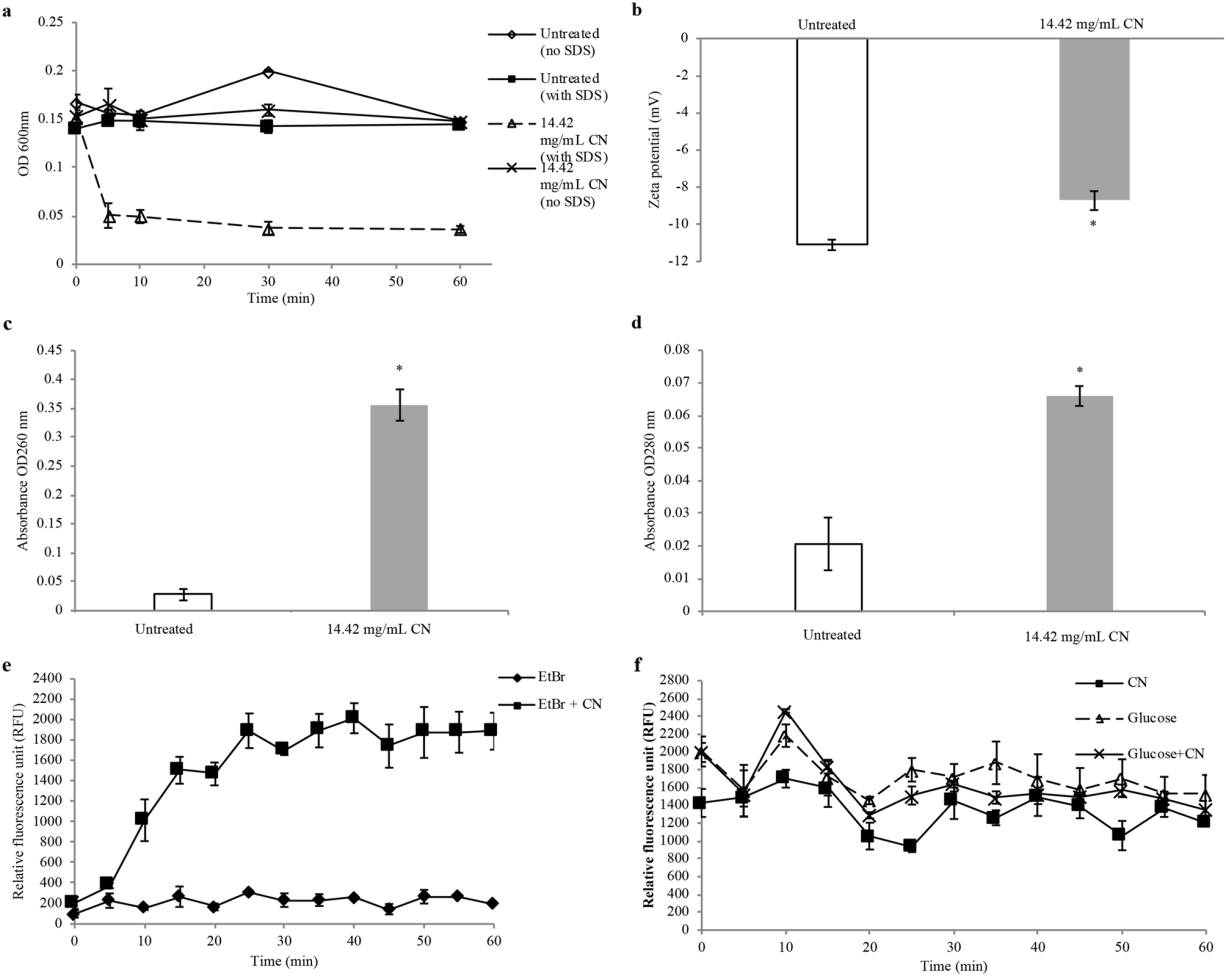
Membrane-related assays performed showing CN disrupts the bacterial membrane of KPC-KP. (**a**) Outer membrane permeability of KPC-KP cells. Comparative absorbance of KPC-KP cells exposed to 0.1% SDS or saline after treatment with CN at 14.42 mg/mL (**b**) Membrane zeta potential of untreated and CN-treated KPC-KP. (**c**) Leakage of UV-absorbing materials; nucleic acids and (**d**) proteins of KPC-KP treated with CN. (**e**) EtBr influx assay with untreated KPC-KP and CN-treated KPC-KP cells exposed to 1 mg/L of EtBr for 60 min at 5 min intervals followed by (**f**) EtBr efflux assay with EtBr removed, treated with glucose or CN only, and combination of glucose with CN. The mean ± SD for three replicates is illustrated. Data were analysed by Student's t-test with *P < 0.05 being significantly different from the untreated KPC-KP cells.

Outer membrane permeability assay was performed to further assess the membrane permeabilisation ability of CN. The cells were divided into two groups, namely the untreated cells and cells treated with 14.42 mg/mL CN (Fig. 2a). Each treatment group was further divided into two groups which were SDS-exposed and non SDS-exposed. SDS at 0.1% was employed as the permeabilising probe, by which it initiates cell lysis when bacterial membrane disorder has reached a critical extent. Non-SDS exposed groups regardless of with or without CN treatment showed a similar trend due to absence of SDS. This trend is also exhibited by the SDS exposed group which is not treated with CN as no changes in cellular permeability was observed. For the group with SDS exposure and treated by CN, there is significant decrease in absorbance indicating reduction in cell viability and increases in SDS permeability into the cell membrane. This indicated the ability of CN, despite not being a bactericidal agent when used alone, in enhancing the SDS-induced cell lysis which slows the cell growth.

### Zeta potential measurement

Zeta potential measurement was performed to evaluate the surface charge of the bacterial cells by detecting the motility of bacterial cells in the presence of an electrophoretic force with defined pH and salt concentrations. The treatment time for CN was 12 h as determined from time-kill analysis. The zeta potential of untreated cells was originally − 11.11 mV while cells treated with 14.42 mg/mL CN resulted in a less negative value of − 8.72 mV (Fig. 2b), indicating the increase in bacterial surface charge.

### Measurement of UV-absorbing materials

The absorbance values obtained from measurement of UV-absorbing materials indicate if there is leakage of intracellular materials such as nucleic acids and proteins due to non-selective pore formation^28^. Based on the results obtained (Fig. 2c,d), CN-treated KPC-KP cells at 260 nm (nucleic acids) and 280 nm (proteins) had an absorbance higher than untreated cells (control). The absorbance value for extracellular nucleic acids in 14.42 mg/mL CN-treated KPC-KP cells had a value of 0.356, higher than the untreated KPC-KP cells with the absorbance value of 0.028 (Fig. 2c). For proteins, CN-treated KPC-KP cells had the absorbance value of 0.066, which was also higher than the untreated KPC-KP cells with the absorbance value of 0.021 (Fig. 2d), demonstrating leakage of intracellular materials caused by CN.

### Ethidium bromide influx/efflux assay

In influx/efflux assay, EtBr, a known efflux pump substrate was used as a probe to determine the membrane permeability and influx inhibiting potential of CN. Cells were treated with EtBr and the compound's concentration was set at sub-MIC to evaluate the accumulation of EtBr. Based on the fluorescence reading, CN-treated KPC-KP (1888 relative fluorescence units, RFU) showed increased in EtBr accumulation level as compared to the untreated cells (200 RFU) by 9.4-fold at the 60 min (Fig. 2e, Table 1). After the influx assay, efflux assay was immediately performed. During efflux assay, glucose was added to act as an energy source to enhance active efflux and reenergize the cells (Machado et al., 2017). CN-treated KPC-KP showed only a slight reduction in fluorescence reading in the efflux assay, with glucose or without glucose throughout the 60 min of efflux assay (Fig. 2f, Table 2).

**Table 1:** Fluorescent readings of influx assay conducted on KPC-KP cells.

Time (min)	Relative fluorescence unit (RFU) (RFU ± SD)	
	EtBr	EtBr + CN
0	98.00 ± 35.57	204.87 ± 59.96
5	228.35 ± 71.18	374.46 ± 25.61
10	155.56 ± 19.25	1015.74 ± 204.31
15	266.67 ± 100.00	1505.09 ± 131.76
20	166.67 ± 33.33	1469.31 ± 112.07
25	311.11 ± 19.25	1892.13 ± 169.29
30	233.33 ± 66.67	1696.30 ± 42.07
35	233.33 ± 57.74	1893.52 ± 164.11
40	255.56 ± 19.25	2015.28 ± 147.32
45	144.44 ± 50.92	1742.01 ± 211.75
50	266.67 ± 66.67	1875.60 ± 249.20
55	266.67 ± 0.00	1877.78 ± 200.92
60	200.00 ± 0.00	1887.96 ± 180.71

**Table 2:** Fluorescent readings of efflux assay conducted on KPC-KP cells.

Time (min)	Relative fluorescence unit (RFU) (RFU ± SD)		
	CN	Glucose	Glucose + CN
0	1427.78 ± 157.53	2005.56 ± 168.60	1994.44 ± 107.15
5	1488.89 ± 101.84	1566.67 ± 288.68	1533.33 ± 260.34
10	1700.00 ± 100.00	2183.33 ± 125.83	2461.11 ± 19.25
15	1594.44 ± 211.70	1711.11 ± 200.92	1827.78 ± 69.39
20	1055.56 ± 150.3083	1455.56 ± 41.94352	1288.89 ± 34.69
25	933.33 ± 57.74	1800.00 ± 132.2876	1511.11 ± 101.84
30	1455.56 ± 208.39	1711.11 ± 153.96	1638.89 ± 38.49
35	1261.11 ± 83.89	1877.78 ± 239.41	1488.89 ± 69.39
40	1500.00 ± 218.58	1694.44 ± 280.05	1533.33 ± 66.67
45	1388.89 ± 133.68	1583.33 ± 236.29	1494.44 ± 50.92
50	1061.11 ± 164.43	1705.56 ± 212.35	1566.67 ± 66.67
55	1372.22 ± 101.84	1527.78 ± 193.17	1477.78 ± 76.98
60	1205.56 ± 38.49	1533.33 ± 208.17	1350.00 ± 100.00

### Measurement of ROS and lipid peroxidation assay

**Figure 3:**
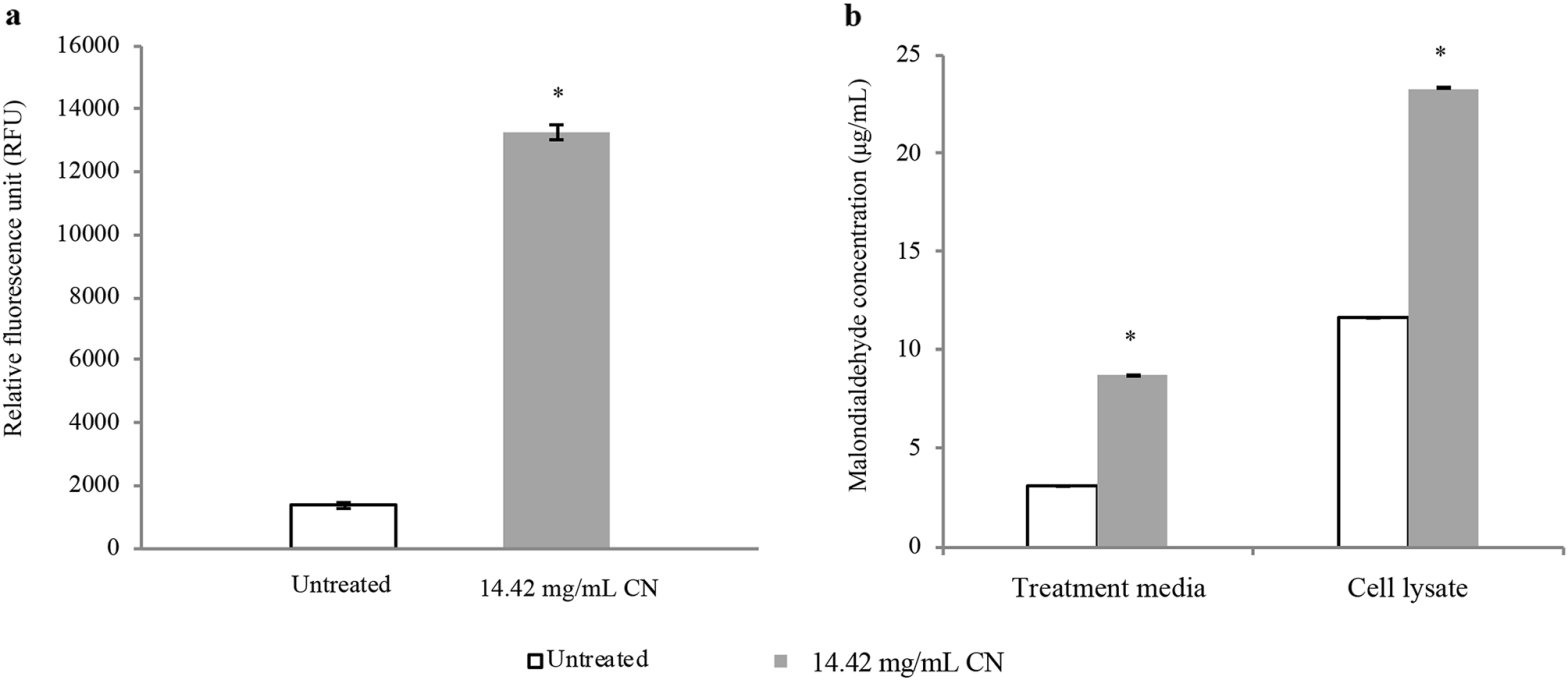
Oxidative stress assessment via (**a**) ROS measurement using DCF-DA and (**b**) lipid peroxidation assay in treated and untreated KPC-KP cells. The mean ± SD for three replicates is illustrated. Data were analysed by Student's t-test with *P < 0.05 being significantly different from the untreated KPC-KP cells.

In order to assess the ability of CN to cause induction of oxidative stress on the KPC-KP cells, the detection of ROS and lipid peroxidation was done by measuring the ROS level and conducting lipid peroxidation assay. Based on the results obtained, the ROS level in CN-treated KPC-KP cells was higher than the untreated cells as shown in Fig. 3a, suggesting more ROS being generated in treated cells than the untreated cells. Furthermore, concentration of MDA was significantly higher in cells exposed to CN compared with the untreated cells (Fig. 3b), demonstrating the presence of lipid peroxidation caused by CN.

### Scanning electron microscopy and transmission electron microscopy

**Figure 4:**
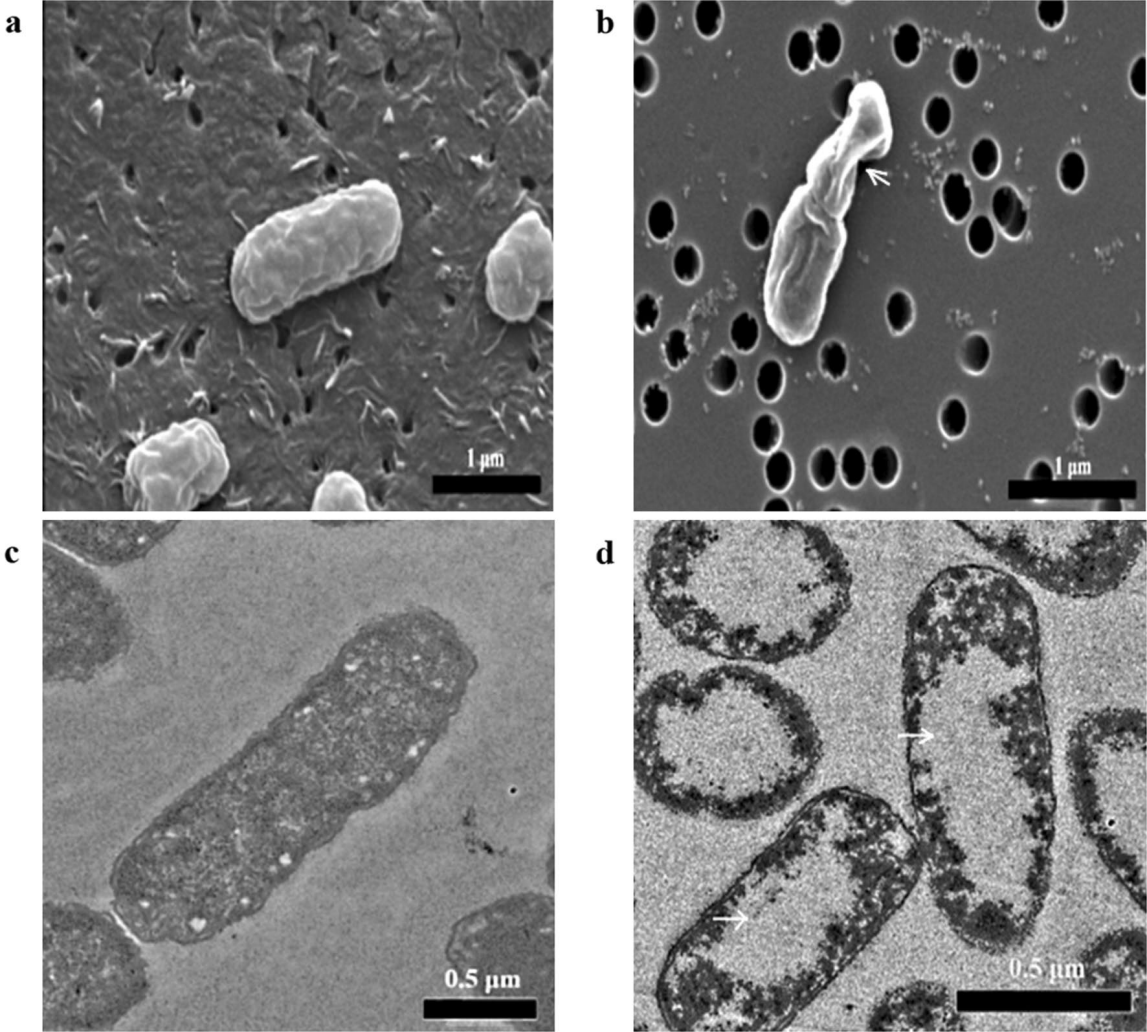
Scanning electron micrographs of KPC-KP. (**a**) Untreated; (**b**) CN-treated KPC-KP cells (14.42 mg/mL). The treated KPC-KP cells with corrugated cell membrane was indicated by the white arrow. Scale bar in scanning electron micrographs represents 1 μm. Tranmission electron micrographs of KPC-KP. (**c**) Untreated; (**d**) CN-treated KPC-KP cells (14.42 mg/mL). The treated cells with cytoplasmic clear zones and lacked of staining zones were indicated by the white arrows. Scale bar in scanning electron micrographs represents 0.5 μm.

Scanning electron microscopy (SEM) and transmission electron microscopy (TEM) were performed to further observe the overall morphology changes in KPC-KP cells after treatment with 14.42 mg/mL CN. The scanning electron micrograph of the untreated KPC-KP cells showed the original morphology (Fig. 4a), which is rod-shaped with smooth surfaces and no observable alterations. However, irregularities had been observed on the surface of the bacterium exposed to CN with corrugated cell membrane. The corrugated cell surface was indicated by the white arrows (Fig. 4b). Upon further inspection via TEM, the untreated cells were normal with full intracellular contents indicating no leakage of intracellular membrane (Fig. 4c), whereas in CN-treated samples, the cells were tremendously affected with apparent cytoplasmic clear zones indicated by the white arrows (Fig. 4d).

## Discussion

In recent years, the rapid rise in antibiotic resistance is a major challenge in the clinical setting. This occurrence prevents patients from recovering, thus increasing the morbidity and mortality rates, especially in immunocompromised patients. Moreover, the emergence of carbapenem-resistant bacteria such as KPC-KP further complicates the mitigation of antibiotic resistance. This situation is especially worrying as carbapenems are the last line β-lactam antibiotics, possessing the widest spectrum of activity and being the most potent amongst all β-lactam antibiotics^7,8^. This serious public health concern could lead to the return of the pre-antibiotic era; therefore, the discovery of new antibiotics is an urgent need. This need has encouraged researchers to look for alternatives such as plant secondary metabolites such as terpenes and terpenoids with hopes of solving the problem. Despite the bioactivity of terpenes and terpenoids has begun documented in several studies, little is known about the mode of action of terpenes against bacteria, especially with reference to antibiotic resistant bacteria. Therefore, this study was performed to investigate the mode of action of CN in killing KPC-KP.

In this study, the antibacterial activity of the monoterpene CN, was first assessed against KPC-KP. The MIC of CN against the tested bacteria was found to be 28.83 mg/mL. In a study reported by Yang et al.^33^ that linalool had a MIC of 11.25 mg/mL against KPC-KP. However, in this study, the MIC of CN reported was 28.83 mg/mL when used against KPC-KP. The higher MIC of CN compared to linalool was due to difference in their structure. CN is a terpenoid ether that lacked of free hydroxyl group, which is present in the alcoholic terpene linalool. The presence of free hydroxyl groups in alcoholic terpenes enables higher potentiating capacity than terpenes devoid of free hydroxyl groups^34^. Compounds or antimicrobials are categorized as bactericidal when there is a reduction of ≥ 3 log10 in CFU/mL whereas reduction of ˂ 3 log10 in CFU/mL is bacteriostatic relative to the initial inoculum concentration^35^. As depicted in the result obtained from time-kill analysis (Fig. 1), KPC-KP cells exposed to CN at half MIC were killed for first 8 h followed by regrowth due to the emergence of resistance. The regrowth phenomenon was due to the favoured killing of the sub-population that is susceptible to CN, together with selective amplification of the resistant sub-population^36^. At MIC, there was reduction of ≥ 3 log10 in CFU /mL number, hence, the antibacterial activity of CN against KPC-KP cells is categorized as bactericidal. As CN is a major component in many essential oils, we postulate that CN may be involved in bacterial membrane disruption. According to several studies, possible mechanisms of essential oils against bacteria are (i) increase in membrane permeability leading to membrane damage; (ii) membrane disruption causing the leakage of cell content and (iii) induction of oxidative stress on bacteria, causing damage on the membrane leading to cell death^31,32^. Hence, we performed quantitative and qualitative membrane-related assays as proof of concept in elucidating the mode of action of CN against KPC-KP.

Bacterial cell surface charge plays an important role in microbial balance and resistance to antimicrobials^37^. The alteration in the zeta potential value, becoming less negative, will lead to increase surface permeability which will eventually decrease cell viability^38,39^. Under normal physiological conditions, the net negative bacterial surface charge is contributed by the carboxyl and phosphate anionic groups in their membranes and is balanced by positively charged counter ions in the surrounding media^39^. The magnitude of the charge differs between species, perhaps due to various culture conditions such as culture age, pH and ionic strength^40^. Based on the result obtained in Fig. 2b, the zeta potential value of CN-treated KPC-KP was less negative in comparison with the untreated KPC-KP cells. This showed that CN had significantly affected the bacterial membrane by increasing the overall surface charge of KPC-KP cells. Similar findings were obtained by Yang et al.^32^ that after exposure to linalyl anthranilate, there was a reduction in the negative surface charge (less negative values) of *K. pneumoniae*, from − 12.1 mV to more positive zeta potential ranging from − 7.53 to − 8.60 mV. The negative surface charge of KPC-KP is related to the abundance of lipopolysaccharides (LPS) that makes up the bacterial outer membrane. Increase in the overall surface charge indicates loss of LPS.

In the outer membrane permeability assay, Gram-negative bacteria with a healthy bacterial membrane will exclude the anionic SDS molecules, due to its negatively charged outer membrane^41^. KPC-KP cells were exposed to SDS at the optimized concentration of 0.1% that does not cause damage to bacterial cells with healthy outer membrane. The viability was evidenced to be declined in CN treated KPC-KP and exposed to SDS at 0.1% (w/v) as shown in Fig. 2a by the significant lower absorbance values compared to cells of other treatment. SDS acts as a permeabilizing probe whereby it only initiates cell lysis when membrane disorder has reached a critical extent^41^. The result indicated that CN disturbed the outer membrane barrier, promoting the influx of SDS into the cells and led to cell lysis. A similar finding was obtained in a study that when KPC-KP cells were treated with a terpene, linalyl anthranilate at 1.25% (v/v), the influx of SDS into the cells occurred, eventually killing them^32^. Despite SDS readily dissolving the cytoplasmic membrane of bacteria, treatment with 0.1% SDS did not show any significant lytic effect as shown in the control without pre-treatment of CN. The duration of this assay was restricted to 60 min in order to prevent any possible cell lysis reaction as a consequence of prolonged exposure to SDS^42^.

In EtBr influx/efflux assay, EtBr was accumulated due to the presence of CN. Glucose was then added to act as an energy source in enhancing active efflux. However, KPC-KP cells did not actively remove EtBr in the efflux assay (Fig. 4b). Although there was accumulation of EtBr during the influx assay in CN-treated KPC-KP cells, the influx could be due to the membrane damage caused by CN. The efflux activity of bacterial cells involves the generation of energy^29^. Besides that, the loss of membrane integrity is highly correlated to the loss of the cell ability to synthesize ATP^43^. As shown in this study, the membrane integrity of KPC-KP was affected by CN based on the change in surface charge as well as the increase in outer membrane permeability of KPC-KP cells. That can impair the respiratory chain functions, resulting in reduced ATP levels. The irreversible cell damage with reduced ATP levels could result in EtBr not being actively removed. In a study performed by Machodo et al., *E. coli* cells treated with 2-phenylquinoline (PQQ4R) at sub-inhibitory concentration (1/8 MIC) resulted in 4% membrane permeation and inhibited the efflux pump. The inhibitory effect was transient when the cells are washed and provided with glucose as the energy source to reenergize the cells and to promote active efflux. However, when *E. coli* cells treated with PQQ4R at half MIC, membrane permeation increased to 28% in tandem with ATP depletion^43^. Hence, EtBr not being actively pumped out from KPC-KP cells could be due to irreversible membrane damage and the depletion of ATP levels. This indicated that CN at half MIC affects the membrane permeability of KPC-KP cells.

Moreover, it was evident in Fig. 2c,d that intracellular nucleic acids and proteins were released into the extracellular environment following the exposure of CN based on the significantly higher absorbance values in the media of CN-treated KPC-KP. This suggests there was non-selective pore formation and disruption in membrane which were observed in the earlier assay, inducing leakage of intracellular materials into the extracellular environment^28^. Similar results have been observed by Yang et al.^32^ whereby KPC-KP cells treated with the compound linalyl anthranilate caused intracellular leakage of nucleic acid and proteins. Zengin and Baysal^18^ reported compounds such as CN, α-terpineol and linalool caused alterations of the outer membrane and leakage of intracellular materials in *S. aureus* and *E. coli* O157:H7. It is crucial to understand that a disrupted cell membrane system may affect other cellular structures in a series of action, such as the lysis of bacterial cell wall, prompting the loss of intracellular dense material^44^. The hypothesis is further confirmed through the scanning electron micrographs. The overall CN-treated bacterial surface is structurally different from that of the untreated KPC-KP cells. The change in cell morphology occurs primarily due to the disruption of membrane structure as evident by the previous findings^31^. A corrugated bacterial membrane was observed in the scanning electron micrograph of CN exposed KPC-KP cells. Guimarães et al.^45^ obtained images by SEM described the death of bacteria treated with carvacrol, l-carveol, eugenol, trans-geraniol, and thymol was due to the loss of cellular membrane integrity and function**.** This result supports the results of other assays that conclude CN interacts with the membrane causing the outer cell membrane disruption. Upon further inspection via TEM, CN-treated samples were significantly affected with apparent cytoplasmic clear zones due to the bacterial membrane damage caused by CN leading to intracellular leakage which was shown in the region devoid of stain (Fig. 4d). De Sousa et al.^46^ observed in the SEM and TEM images of CN-treated *Aeromonas hydrophila* had a damaged cell envelope and leakage of intracellular contents. The result of TEM supports the result obtained from the measurement of UV-absorbing materials assay indicating CN disrupts the KPC-KP membrane causing leakage of intracellular materials.

Formation of ROS in bacteria is also part of the bactericidal activity of antimicrobials. Findings by Hong et al.^47^ revealed that *E. coli* subjected to antimicrobial stress (ampicillin, nalidixic acid and trimethoprim) and subsequently removal of the antimicrobial led to the accumulation of ROS followed by cell death due to post-stress ROS-mediated toxicity. Induction of oxidative stress affects the eukaryotic cellular membrane integrity. This is known as lipid peroxidation, a self-propagating chain reaction involving reactions between ROS and membrane fatty acid, eventually destroying cell membrane^48,49^. In this study, the detection of ROS and lipid peroxidation was done by measuring the ROS level and conducting lipid peroxidation assay. The stress condition of the cells is usually defined by the level of ROS and MDA^31^. Higher level of ROS and MDA in cells exposed to CN than the untreated KPC-KP cells as seen in Fig. 2e,f demonstrated the presence of lipid peroxidation caused by CN. Linalyl anthranilate, a terpene found in a variety of plants such as marjoram, lavender and thyme induce oxidative stress in KPC-KP cells by reacting with bacterial membrane component^32^. The reaction produces ROS, which attacks the membrane lipids causing a chain reaction finally disrupting the bacterial membrane; this is termed as lipid peroxidation allowing the influx of ROS into the KPC-KP intracellular region. ROS degrades lipids, nucleic acids and proteins. Ultimately, intracellular materials leak out from the cells due to bacterial membrane disruption^31^. Besides that, molecular dynamics simulations, a technique used to probe interaction of small molecules with model lipid membranes, have shown that terpenes such as perillyl derivatives (perillyl alcohol, acid and aldehyde) and limonene interact and perforate the membrane^50^. The terpene molecules then interact with phosphate groups of the lipids and the glycerol backbone, and perillic acid that is negatively charged interacts with choline of the head group. For limonene, after membrane penetration, it is able to stay at the bilayer center as it has no polar substituents^50,51^. It appears that plant-derived molecules disintegrate membranes, some make the membranes become more fluid or stabilize and order them^52^. The study of molecular dynamics simulations of terpenes with lipid bilayer membranes together with experimental investigations in this study is in support with the general picture that terpenes act by altering the physical properties of the lipid bilayer components of the membranes.

**Figure 5:**
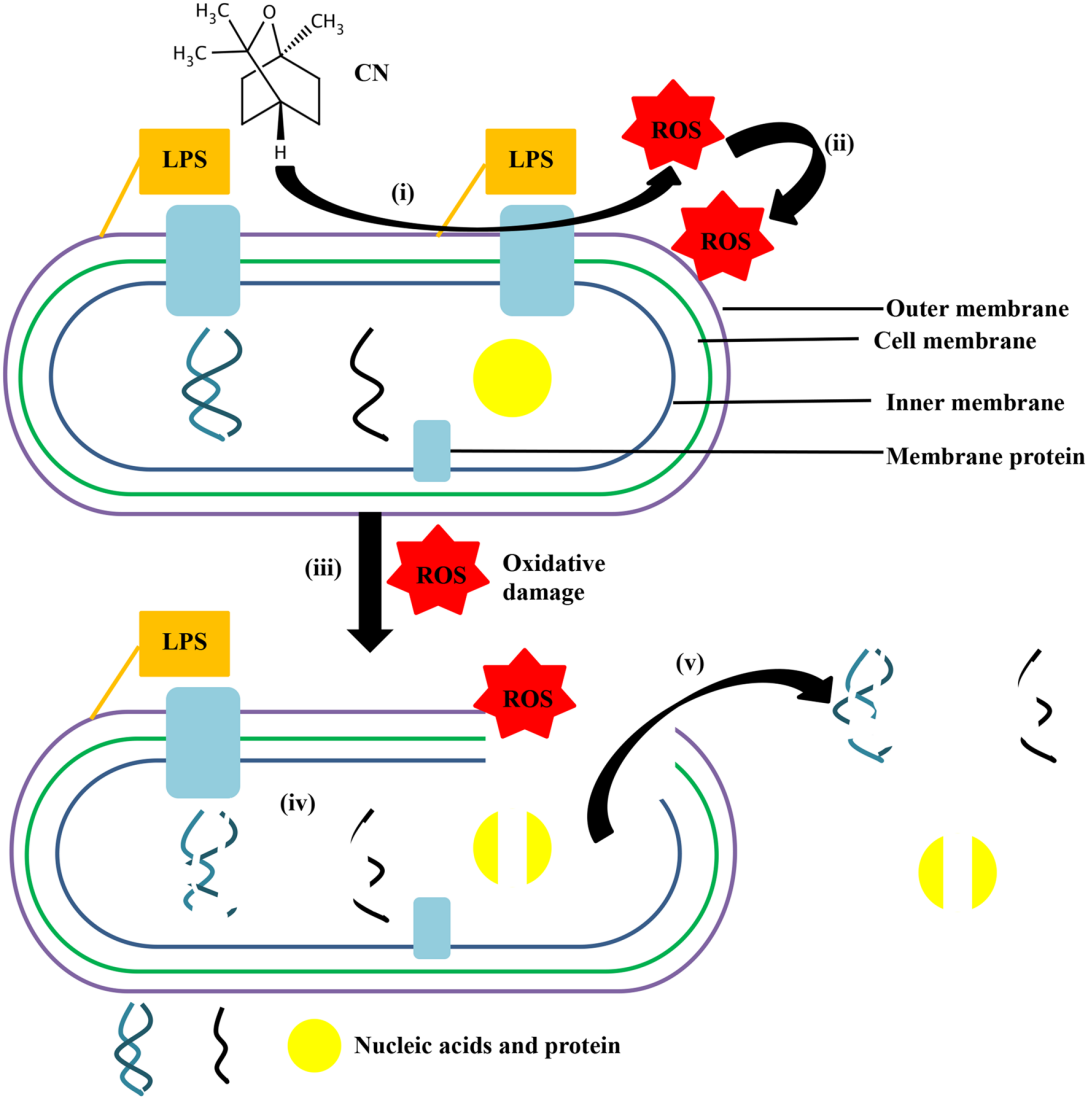
The proposed mode of action of CN against KPC-KP cells. (**i**) CN reacted with the bacterial membrane which produced ROS. (**ii**) ROS attacks membrane lipid to initiate lipid peroxidation, causing chain reaction which disrupts the bacterial membrane. (**iii**) The disrupted membrane leads to the influx of ROS into the intracellular region of KPC-KP. (**iv**) ROS degrades intracellular materials such as nucleic acids, proteins and lipids. (**v**) Intracellular materials leak out due to membrane disruption.

In conclusion, this study reported the antibacterial activity and the mode of action of CN against KPC-KP. CN induced oxidative stress led to the disruption in bacterial membrane via lipid peroxidation and intracellular materials leakage, and consequently causing cell death. The proposed mode of action of CN is illustrated in Fig. 5.

Since the membrane disruption effects of CN are demonstrated, their potential effect on the up or down-regulation of the proteins related to bacterial membrane could be studied further using liquid chromatography-mass spectrometry, as well as study on how CN affecting the bacterial membrane permeability changes porins. In order to truly apply these compounds as therapeutics, toxicology evaluation of the CN and in vivo studies using mouse models should be performed. The determined concentration in toxicology evaluation will serve as a benchmark for in vivo animal studies which is essential prior to clinical trials and eventually, approval. The data suggest that CN is a potential source of alternative to conventional antibiotics for treatment of KPC-KP infections (Supplementary Information).

## Supplementary Information

Supplementary Information.

## Data Availability

All data generated or analysed during this study are included in the Supplementary Information files.
