# Supplementary material for: Antimicrobial activity and mode of action of 1,8-cineol against carbapenemase-producing Klebsiella pneumoniae
Source: Sci Rep. 2021 Oct 21;11:20824. doi: 10.1038/s41598-021-00249-y (PMC8531306; doi:10.1038/s41598-021-00249-y)
Supplement: Supplementary file 1 — Supplementary Information. [file 41598_2021_249_MOESM1_ESM.docx]

**Antimicrobial activity and mode of action of 1,8-cineol against carbapenemase-producing *Klebsiella pneumoniae***

**Chew-Li Moo^1^, Mohd Azuraidi Osman^1^, Shun-Kai Yang^2^, Wai-Sum Yap^3^, Saila Ismail^4^, Swee-Hua-Erin Lim^2^, Chou-Min Chong^5*^, Kok-Song Lai^2*^**

^1^Department of Cell and Molecular Biology, Faculty of Biotechnology and Biomolecular Sciences, Universiti Putra Malaysia, 43400 Serdang, Selangor, Malaysia.

^2^Health Sciences Division, Abu Dhabi Women's College, Higher Colleges of Technology, 41012 Abu Dhabi, United Arab Emirates.

^3^Faculty of Applied Sciences, UCSI University, No. 1, Jalan Menara Gading UCSI Height, 56000 Cheras, Kuala Lumpur, Malaysia.

^4^Department of Microbiology, Faculty of Biotechnology and Biomolecular Sciences, Universiti Putra Malaysia, 43400 Serdang, Selangor, Malaysia.

^5^Aquatic Animal Health and Therapeutics Laboratory, Institute of Bioscience, Universiti Putra Malaysia, Serdang 43400, Selangor, Malaysia.

**Supplementary material**

**Supplementary Table S1** Time-kill analysis data KPC-KP cells treated with CN

| Time(hour) | KPC-KP cell number (CFU/mL) (Mean ± SD) | | |
| --- | --- | --- | --- |
|  | Untreated | CN (3.13 %) | CN (1.56 %) |
|  |  |  |  |
| 0 | 4.99 ± 0.01 | 5.56 ± 0.07 | 4.71 ± 0.06 |
| 2 | 5.00 ± 0.00 | 0 ± 0 | 0 ± 0 |
| 4 | 7.86 ± 0.20 | 0 ± 0 | 0 ± 0 |
| 8 | 10.16 ± 0.01 | 0 ± 0 | 0 ± 0 |
| 12 | 10.16 ± 0.01 | 0 ± 0 | 3.87 ± 0.00 |
| 16 | 10.80 ± 0.17 | 0 ± 0 | 7.90 ± 0.17 |
| 20 | 10.70 ± 0.00 | 0 ± 0 | 8.67 ± 0.06 |
| 24 | 10.90 ±0.17 | 0 ± 0 | 9.1 ± 0.00 |

**Supplementary Table S2** Zeta potential measurement data of KPC-KP cells treated with CN.

|  | Zeta potential (mV) (Mean ± SD) | |
| --- | --- | --- |
| KPC-KP | **Untreated** | **CN (1.56 %)** |
|  | -11.11 ± 0.28 | -8.72 ± 0.51 |

**Supplementary Table S3** Raw data of influx/efflux assay of KPC-KP.

**Influx**

|  | Relative fluorescence unit (RFU) (RFU ± SD) | |
| --- | --- | --- |
| Time (min) | **EtBr** | **EtBr + CN** |
| 0 | 98.00 ± 35.57 | 204.87 ± 59.96 |
| 5 | 228.35 ± 71.18 | 374.46 ± 25.61 |
| 10 | 155.56 ± 19.25 | 1015.74 ± 204.31 |
| 15 | 266.67 ± 100.00 | 1505.09 ± 131.76 |
| 20 | 166.67 ± 33.33 | 1469.31 ± 112.07 |
| 25 | 311.11 ± 19.25 | 1892.13 ± 169.29 |
| 30 | 233.33 ± 66.67 | 1696.30 ± 42.07 |
| 35 | 233.33 ± 57.74 | 1893.52 ± 164.11 |
| 40 | 255.56 ± 19.25 | 2015.28 ± 147.32 |
| 45 | 144.44 ± 50.92 | 1742.01 ± 211.75 |
| 50 | 266.67 ± 66.67 | 1875.60 ± 249.20 |
| 55 | 266.67 ± 0.00 | 1877.78± 200.92 |
| 60 | 200.00 ± 0.00 | 1887.96 ± 180.71 |

**Efflux**

|  | Relative fluorescence unit (RFU) (RFU ± SD) | | |
| --- | --- | --- | --- |
| Time (min) | **CN** | **Glucose** | **Glucose + CN** |
| 0 | 1427.78 ± 157.53 | 2005.56 ± 168.60 | 1994.44 ± 107.15 |
| 5 | 1488.89 ± 101.84 | 1566.67 ± 288.68 | 1533.33 ± 260.34 |
| 10 | 1700.00 ± 100.00 | 2183.33 ± 125.83 | 2461.11 ± 19.25 |
| 15 | 1594.44 ± 211.70 | 1711.11 ± 200.92 | 1827.78 ± 69.39 |
| 20 | 1055.56 ± 150.3083 | 1455.56 ± 41.94352 | 1288.89 ± 34.69 |
| 25 | 933.33 ±57.74 | 1800.00 ± 132.2876 | 1511.11 ±101.84 |
| 30 | 1455.56 ± 208.39 | 1711.11 ± 153.96 | 1638.89 ± 38.49 |
| 35 | 1261.11 ± 83.89 | 1877.78 ± 239.41 | 1488.89 ± 69.39 |
| 40 | 1500.00 ± 218.58 | 1694.44 ± 280.05 | 1533.33 ± 66.67 |
| 45 | 1388.89 ± 133.68 | 1583.33 ± 236.29 | 1494.44 ± 50.92 |
| 50 | 1061.11 ± 164.43 | 1705.56 ± 212.35 | 1566.67 ± 66.67 |
| 55 | 1372.22 ± 101.84 | 1527.78 ± 193.17 | 1477.78 ± 76.98 |
| 60 | 1205.56 ± 38.49 | 1533.33 ± 208.17 | 1350.00 ± 100.00 |

**Supplementary Table S4** UV-absorbing materials measurement data of KPC-KP cells treated with CN.

**Nucleic acids**

|  | Absorbance OD 260 nm (Mean ± SD) | |
| --- | --- | --- |
| KPC-KP | **Untreated** | **CN (1.56 %)** |
|  | 0.028 ± 0.010 | 0.356 ± 0.027 |

**Proteins**

|  | Absorbance OD 280 nm (Mean ± SD) | |
| --- | --- | --- |
| KPC-KP | **Untreated** | **CN (1.56 %)** |
|  | 0.021 ± 0.008 | 0.066 ± 0.003 |

**Supplementary Table S5** Raw data of outer membrane permeability assay

|  | Untreated | | CN-treated (1.56 %) | |
| --- | --- | --- | --- | --- |
| Time (min) | **With 0.1 % SDS** | **Without 0.1 % SDS** | **With 0.1 % SDS** | **Without 0.1 % SDS** |
| 0 | 0.140 ± 0.004 | 0.167 ± 0.008 | 0.152 ± 0.010 | 0.153 ± 0.004 |
| 5 | 0.148 ± 0.004 | 0.156 ± 0.004 | 0.050 ± 0.013 | 0.166 ± 0.016 |
| 10 | 0.148 ± 0.010 | 0.154 ± 0.004 | 0.049 ± 0.007 | 0.150 ± 0.008 |
| 30 | 0.142 ± 0.007 | 0.200 ± 0.001 | 0.037 ± 0.007 | 0.159 ± 0.006 |
| 60 | 0.144 ± 0.004 | 0.148 ± 0.000 | 0.036 ± 0.004 | 0.148 ± 0.000 |

**Supplementary Table S6** Lipid peroxidation assay data of KPC-KP cells treated with CN.

|  | Malondialdehyde (μg/mL ± SD) | |
| --- | --- | --- |
|  | **Untreated** | **CN-treated (1.56 %)** |
| Treatment media | 3.122 ± 0.007 | 8.744 ± 0.027 |
| Cell lysate | 11.656 ± 0.003 | 23.356 ± 0.067 |

**Supplementary Table S7** ROS level measurement data of KPC-KP cells treated with CN.

|  | Relative fluorescence unit (RFU) (RFU ± SD) |
| --- | --- |
| Untreated | 1380.111 ± 93.22881 |
| CN-treated (1.56 %) | 13261.33 ± 238.5666 |
